# Closing the gap in plan quality: Leveraging deep‐learning dose prediction for adaptive radiotherapy

**DOI:** 10.1002/acm2.70045

**Published:** 2025-03-19

**Authors:** Sean J. Domal, Austen Maniscalco, Justin Visak, Michael Dohopolski, Dominic Moon, Vladimir Avkshtol, Dan Nguyen, Steve Jiang, David Sher, Mu‐ Han Lin

**Affiliations:** ^1^ Department of Radiation Oncology University of Texas Southwestern Medical Center Dallas Texas USA; ^2^ Medical Artificial Intelligence and Automation Laboratory Department of Radiation Oncology University of Texas Southwestern Medical Center Dallas Texas USA

**Keywords:** adaptive therapy, AI, Ethos, head and neck

## Abstract

**Purpose:**

Balancing quality and efficiency has been a challenge for online adaptive therapy. Most systems start the online re‐optimization with the original planning goals. While some systems allow planners to modify the planning goals, achieving a high‐quality plan within time constraints remains a common barrier. This study aims to bolster plan quality by leveraging a deep‐learning dose prediction model to predict new planning goals that account for inter‐fractional anatomical changes.

**Methods:**

Fine‐tuned patient‐specific (FT‐PS) models were clinically evaluated to accurately predict dose for 23 adaptive fractions of 15 head‐and‐neck (H&N) patients treated with Ethos ART. The original adapted plan from the adaptive treatment session was used as the quality baseline. Based on physician‐approved adaptive treatment contours, the FT‐PS model predicted subsequent planning goals for high‐impact organs at risk (OARs). These goals were retrospectively re‐optimized in Ethos to compare the original adapted plan (IOE‐Auto Plan) with the newly re‐optimized plan (AI‐guided IOE Plan). A physician blindly selected the preferred plan.

**Results:**

Dose savings were observed for nine high impact OAR's including the constrictor, ipsilateral/contralateral parotid, ipsilateral/contralateral submandibular gland, oral cavity, and esophagus, mandible and larynx with a maximum value of 5.47 Gy. Of the 23 plans reviewed in the blind observer study, 19 re‐optimized plans were chosen over the original adapted session plan.

**Conclusions:**

Our preliminary results demonstrate the feasibility of utilizing an AI dose predictor to predict optimal planning goals with anatomical changes, thereby improving adaptive plan quality. This method is feasible for both online and offline adaptive radiotherapy (ART) and has the potential to significantly enhance treatment outcomes for head‐and‐neck (H&N) cancer patients.

## 1 INTRODUCTION

Radiotherapy plays a vital role in the comprehensive treatment of head and neck (HN) cancer, often complementing other modalities like surgery and chemotherapy.^1^, ^2^, ^3^ Traditionally, 3D conformal RT (3DCRT) has been the standard, but recent advances in radiotherapy techniques, particularly intensity‐modulated radiotherapy (IMRT), and volumetric modulated arc therapy (VMAT), have ushered in an era of remarkable precision.^4^, ^5^ These sophisticated approaches allow clinicians to precisely target radiation, concentrating on the tumor while minimizing exposure to surrounding healthy tissues. This refined precision not only translates into improved treatment outcomes but also can reduce adverse side effects commonly associated with HN cancer therapies. Furthermore, IMRT and VMAT facilitate the development of multi‐level simultaneous integrated boost (SIB) treatment plans.^4^, ^5^

Adaptive radiotherapy (ART) is a technique that has emerged in recent years to further improve the precision and accuracy of radiation therapy for cancer treatment.^6^ This may especially prove advantageous for the management of HN cancers.^7^ Traditional radiation therapy involves creating a treatment plan based on a single imaging scan, such as a CT or MRI, which is used to deliver a fixed dose of radiation over the course of treatment. However, as the tumor and surrounding tissues can change shape and size over time due to factors such as tumor shrinkage or swelling, weight loss or gain, and movement of nearby organs, this fixed treatment plan may not be optimal throughout the course of treatment. Offline ART involves modifying the treatment plan between treatment sessions based on imaging and other data collected prior to treatment. This method of ART can present challenges such as the need for accurate pre‐treatment imaging, ensuring the stability of the tumor and surrounding tissues between treatments. However, offline ART may be more suitable for tumors located in organs that do not move significantly.^6^, ^8^ Conversely, online ART entails revising the treatment plan at the beginning of the treatment session. This adjustment is based on the most recent imaging and data, collected while the patient is positioned on the treatment table, to ensure the therapy is accurately tailored based on the anatomy of the day. This approach can present several challenges, such as the need for frequent imaging and data collection, treatment plan modification, quality assurance, resource allocation, data analysis, and interpretation. However, online ART has the advantage of addressing tumor changes as they occur and can be particularly useful for tumors located in organs that move.^6^, ^8^

The Ethos system by Varian Medical Systems incorporates artificial intelligence (AI) and GPU‐based calculation engines to expedite the ART workflow, potentially offering efficiency improvements over traditional methods. The adaptive features of the Ethos system, including AI‐assisted contouring and a streamlined adaptive workflow, are extensively documented in existing literature and have been successfully employed by diverse institutions to treat a wide range of tumor sites.^9^, ^10^, ^11^, ^12^, ^13^ The system has proven particularly effective in treating head and neck (HN) cancers, showcasing the potential benefits of integrating advanced AI and streamlined workflows in cancer treatment.^9^, ^14^

While systems like Ethos demonstrate considerable progress in online ART with their speed and efficiency, it's important to recognize a common limitation in many ART systems: their reliance on static treatment objectives based on the patient's initial planning CT scan. This static approach may not adequately accommodate significant anatomical changes that can occur during the treatment course. Therefore, to fully realize the potential of these systems, it's critical to address this limitation, especially in response to dynamic changes in patient anatomy. This issue of a static treatment approach is underscored by recent findings from a phase 3 randomized clinical trial, which reported less favorable results in the context of adaptive planning.^15^ A key observation from the trial was the use of similar optimization objectives for both initial and adaptive plans, which may have restricted the ability to thoroughly spare critical structures. This insight underscores the pressing need for innovative solutions, such as AI to dynamically guide users in adapting planning objectives to ensure the production of optimal treatment plans and, consequently, the best possible patient outcomes.

In recent years we have seen the integration of AI into ART. These advanced techniques have significantly enhanced the efficiency and precision of key functions in ART encompassing tasks such as automating organ segmentation, predicting radiation dose, and generating treatment plans.^13^, ^16^, ^17^ Of particular interest is the concept of dose prediction as a guide for aiding plan creation. Studies underscore AI's efficacy in enhancing reference planning through deep‐learning methodologies, as seen in Visak et al.’s evaluation of planning approaches within the Ethos system.^12^ Their findings favor AI‐guided plans over traditional methods, citing improved target coverage and organ‐at‐risk (OAR) sparing. In a parallel inquiry, Nguyen et al. employed a novel deep learning framework, the hierarchically densely connected U‐net, for anticipating dose distributions in HN cancer case, achieving notable accuracy over prevailing prediction models.^18^ Building on this, Maniscalco et al. pursued a streamlined, patient‐specific approach, necessitating only initial treatment data to inform their model. This approach has exciting potential for optimizing online adaptive RT workflows. Their research, encompassing comprehensive patient data analysis, confirmed the plausibility of using AI‐ integrated techniques for adaptive dose predictions.^19^

**FIGURE 1:**
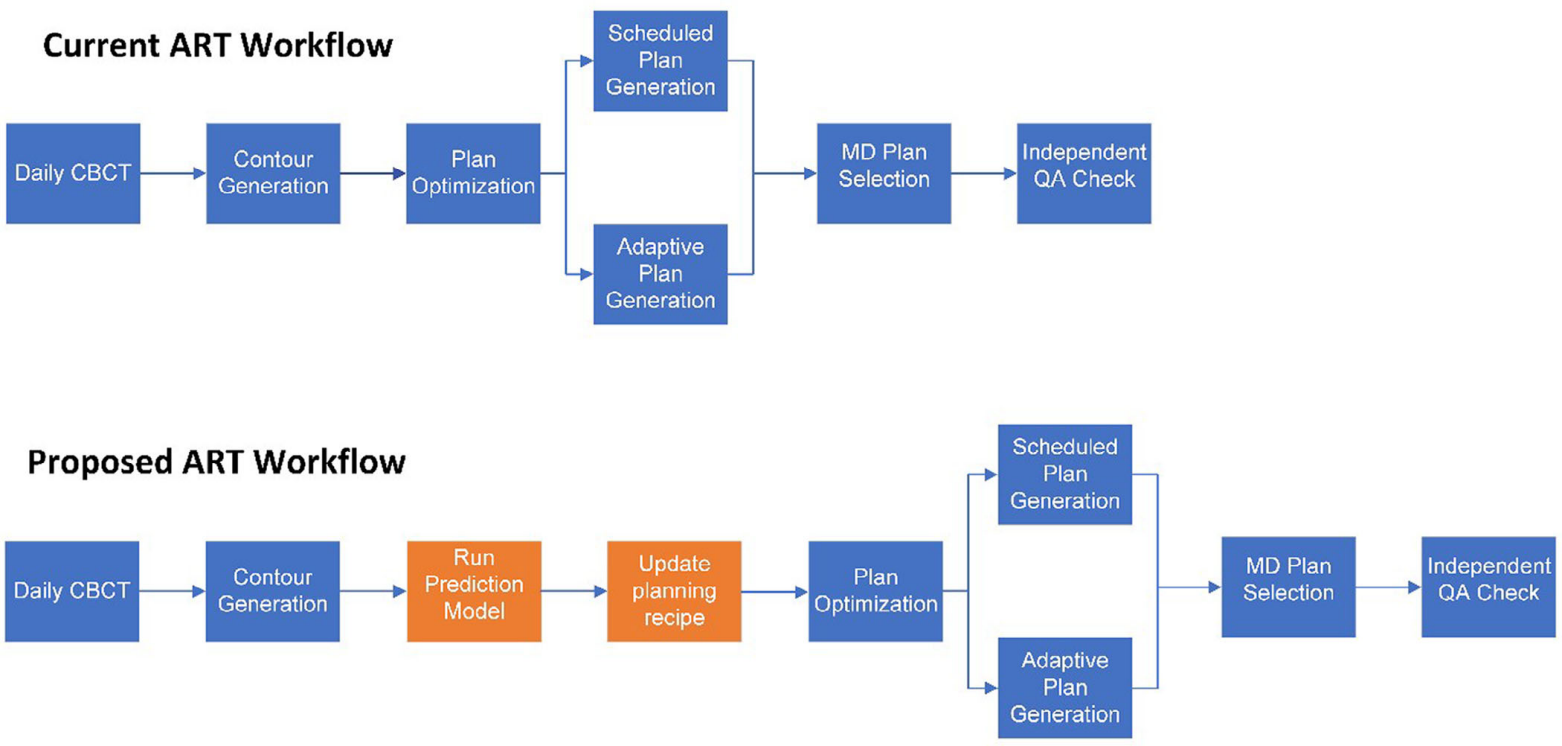
Current versus proposed ART workflow diagram.

By leveraging a previously developed AI‐based dose prediction model and fine‐tuning architecture, this study seeks to overcome the limitation of a static recipe for online re‐optimization^18^, ^19^. Our proposed workflow is to incorporate AI dose prediction in online adaptive workflow to automatically predict the optimal plan quality based on the anatomy of the day and utilize the predicted planning goals to re‐optimize the plan. Figure 1, visually depicts our current ART workflow alongside the proposed ART workflow of this study. We validated our concept using the Ethos system, focusing on head and neck (HN) cancers, which are among the most challenging targets for online adaptive therapy. However, this methodology can be applied to other adaptive therapy systems and treatment sites to bridge the gap between efficiency and optimal plan quality.

## 2 MATERIALS AND METHODS

### 2.1 Model implementation and usage

**FIGURE 2:**
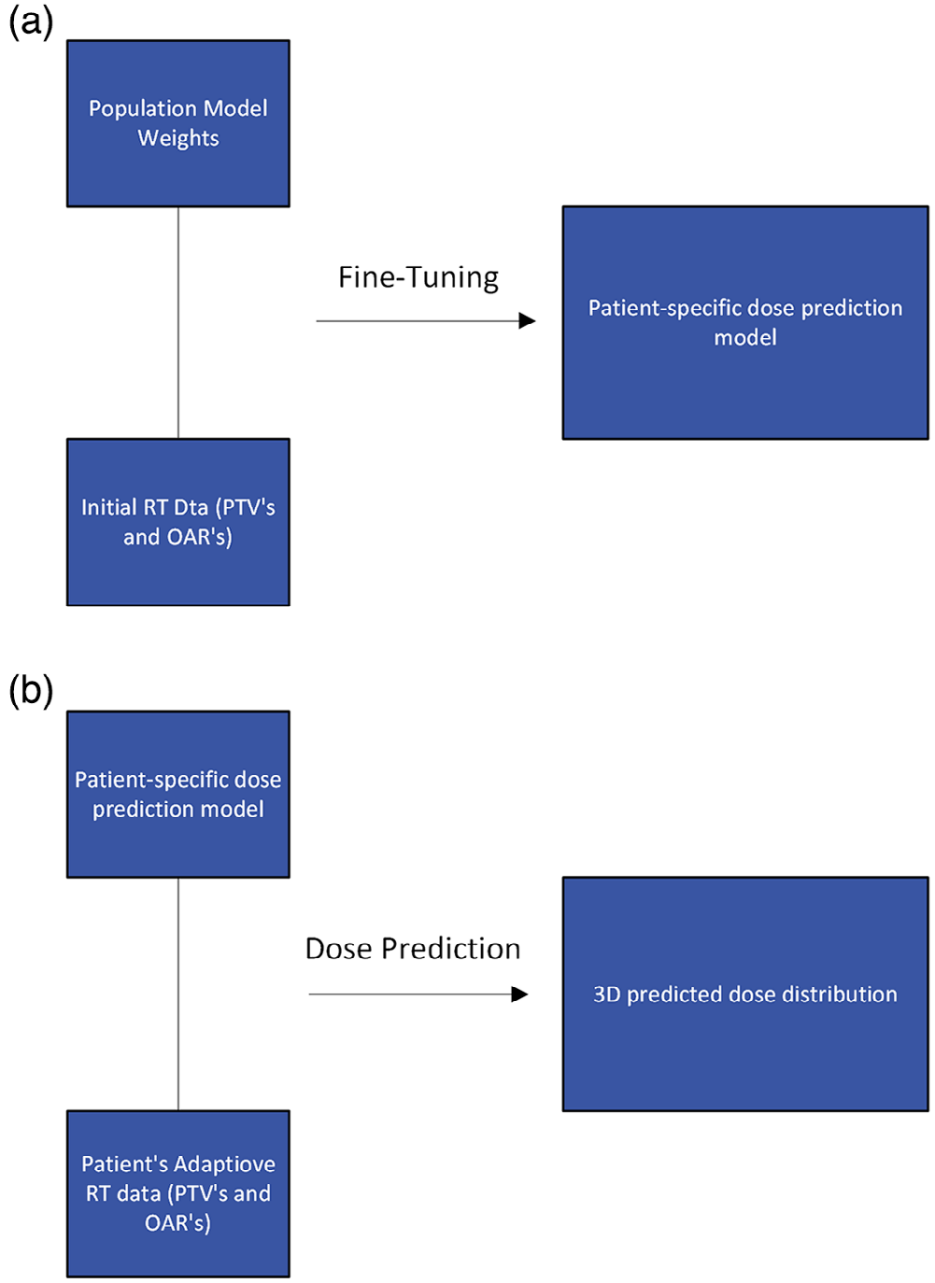
Model fine‐tuning and dose prediction workflow. 2A shows the base model weights and the RT Data from an initial plan which serves as inputs for model fine‐tuning which results in a patient specific dose prediction model. 2B illustrates how this patient specific dose prediction model can be fed RT data from an adaptive session which can produce a 3D predicted dose distribution for a specific adaptive session.

In this study, we employ the population‐based dose prediction model as published by Maniscalco et al., which uses the exact Hierarchically Densely Connected U‐Net architecture as published by Nyguen et al., as base weights to fine‐tune models for individual patients. This results in a distinct patient‐specific model for each individual adaptive case of this study.^19^ As these are promising tools with clinical potential, the aim of this work was to leverage these tools and clinically assess their performance and feasibility for ART. For each patient in this study, we utilized the population‐based model as base model weights and then fine‐tuned the model weights with a single patient's pre‐treatment data. We followed Maniscalco et al.’s hyperparameters, using a batch size of 5, with 10 steps per epoch for a total of 500 epochs. The fine‐tuning process was implemented in Python, utilizing TensorFlow v2.0, where weights from the pre‐trained population model directly from Maniscalco et al. were loaded and trained further using a single patient‐specific data which include PTVs and OARs. The model fine‐tuning process utilized a high‐performance computing cluster at UT Southwestern, where a single NVIDIA V100 graphics processing unit (GPU) with 32GB of VRAM was employed. This GPU served as the computational resource for this work. The resulting model, which is referred to as a fine‐tuned, patient‐specific (FT‐PS) model, was used to predict dose distributions for adaptive sessions based on an adaptive session's PTVs and OARs. Figure 2 displays a diagram of these processes as they were used in this study. This is the exact approach taken by Maniscalco et al. and was kept the same in order to appropriately assess this approach clinically. We evaluated performance in terms of max and mean dose differences across pertinent OARs, which is the most clinically relevant metric for this purpose. It is important to note that the work presented in both the Maniscalco et al. and Nyguen et al. focus exclusively on Head and Neck cancer patients. This work also exclusively focuses on head and neck cancer patients but with a more specific focus on the feasibility of using these tools during adaptive radiotherapy. An obvious advantage of fine‐tuning the population‐based dose prediction model with a patient's pre‐treatment data, the model's weights can be adjusted based on this plan's unique dosimetric tradeoffs. The FT‐PS model has the capacity to predict dose with prior knowledge of desired dosimetric tradeoffs while considering the daily changes in PTVs and OARs, and thereby assists in improving the consistency of plan quality throughout a patient's adaptive treatment. For example, it may predict progressively reduced OAR doses as a patient's target volume shifts away from these critical structures during ART. Additionally, these predictions may help reduce subjectivity and inter‐observer variability in treatment planning, making it a valuable tool for guiding re‐optimization.

### 2.2 Patient selection

This study focused exclusively on HN cancer patients adapted at UTSW medical center on the Varian Ethos system at UT Southwestern Medical Center. The patient selection for this study consisted of participants enrolled in the DARTBOARD clinical trial, which represents the first randomized study of daily adaptive radiation therapy in HN cancer.^20^ Patients enrolled in the DARTBOARD trial required a diagnosis of oropharyngeal, laryngeal, or hypopharyngeal squamous cell carcinoma (SqCC) undergoing definitive radiotherapy (RT) or chemoradiotherapy (CRT). Additionally, adapt on demand patients treated at USTW medical center were included, who underwent multiple adaptations during their treatment based on the clinical judgment of the physicians. The last remaining criteria was that selected patients must not have been a part of the study published by Maniscalco et al to avoid any confounding study results. These patient groups represented most adaptive head and neck (HN) patients being treated with the Ethos system at the time of our study, which is the primary reason for their inclusion. The dose schema for these patients were all SIB with either 2 or 3 dose levels. In the initial selection, a cohort of 21 patients undergoing a total of 31 treated fractions fit the above criteria for analysis using a fine‐tuned patient‐specific (FT‐PS) model. However, for the purpose of this study, only a subset of 15 patients and 23 treated fractions were included. This selection was made because only these 15 patients and 23 fractions exhibited significant enough differences between the original planned organ‐at‐risk (OAR) doses and the AI‐predicted OAR doses to justify re‐optimization. This criterion was critical to ensuring that the study focused on cases where the potential impact of AI guided re‐planning would be most meaningful and observable. Table 1 details the specifics of each patient and adaptive fraction considered in this study.

**TABLE 1:** Patient Specifics.

Patient number	RT intent	Planned fractions	Dose levels	Highest dose level	Adaptive classification	Adapted session
Patient 1	Oropharynx Bilateral	35	3	7000	Daily	18
Patient 1	Oropharynx Bilateral	35	3	7000	Daily	35
Patient 2	Oropharynx Bilateral	35	3	7000	Daily	17
Patient 2	Oropharynx Bilateral	35	3	7000	Daily	34
Patient 3	Oropharynx Bilateral	35	3	7000	Daily	35
Patient 4	Oropharynx Bilateral	35	3	7000	Daily	34
Patient 5	Oropharynx Bilateral	35	3	7000	Daily	18
Patient 5	Oropharynx Bilateral	35	3	7000	Daily	35
Patient 6	Head and Neck Bilateral	35	2	5950	On Demand	12
Patient 7	Oropharynx Bilateral	35	2	7000	On Demand	1
Patient 8	Oropharynx Bilateral	35	2	7000	On Demand	28
Patient 8	Oropharynx Bilateral	35	2	7000	On Demand	18
Patient 8	Oropharynx Bilateral	35	2	7000	On Demand	7
Patient 9	Head and Neck Bilateral	35	3	7000	On Demand	20
Patient 9	Head and Neck Bilateral	35	3	7000	On Demand	11
Patient 10	Oropharynx Left	33	3	6996	On Demand	32
Patient 10	Oropharynx Left	33	3	6996	On Demand	23
Patient 11	Oropharynx Bilateral	29	3	5800	On Demand	11
Patient 12	Oropharynx Bilateral	35	3	7000	On Demand	22
Patient 12	Oropharynx Bilateral	35	3	7000	On Demand	11
Patient 13	Head and Neck Bilateral	35	3	7000	On Demand	30
Patient 14	Oropharynx Bilateral	33	3	6996	On Demand	30
Patient 15	Oropharynx Bilateral	30	3	6360	On Demand	26

*Note*: The fifteen patients and accompanying 23 fractions are shown below. For each patient the RT intent, number of planned fractions, number of dose levels, highest dose level, adaptive classification, and the adaptive fraction considered in this study are presented.

### 2.3 Study design and workflow

The Ethos Adaptive Radiation Therapy (ART) treatment workflow is traditionally carried out using a series of steps tailored to individual patients. This includes:

1. Daily CBCT Acquisition: Each day, a Cone Beam Computed Tomography (CBCT) scan is performed to capture the patient's current anatomical information.
2. Contour Generation: AI algorithms or deformable methods automatically generate influencer contours. Building upon the influencer map, precise contours for the targets and OARs are generated and reviewed by physicians.
3. Recalculation and Re‐optimization: The reference plan derived from the initial CT simulation is recalculated, taking into account the current anatomical information. A new adaptive plan is also re‐optimized using the pre‐established planning goals determined in the original treatment plan.
4. Plan Selection: The physician selects between recalculated or re‐optimized for the patient.
5. Independent Quality Assurance (QA) Check.

For this study, we propose updating the original planning goals with the model's dose predictions before re‐optimizing the plan, aiming to enhance the quality of plans for these adaptive patients. Specifically, this means adjusting the numeric planning goals to align with the values provided by the AI dose prediction. It is important to clarify that this approach does not involve introducing new goals or altering the priority of existing goals during the re‐optimization process; rather, it focuses solely on modifying the numeric values of the existing planning goals to reflect the AI‐generated predictions. To evaluate the effectiveness of this approach, the study retrospectively re‐optimized the selected treatment plans using the Ethos system. A comparison was made between the original adapted plan, known as the IOE‐Auto Plan, and the newly re‐optimized plan guided by AI, referred to as the AI‐guided IOE Plan. The primary objective was to assess whether the AI‐guided plan demonstrated improved quality and efficacy compared to the original adapted plan.

**FIGURE 3:**
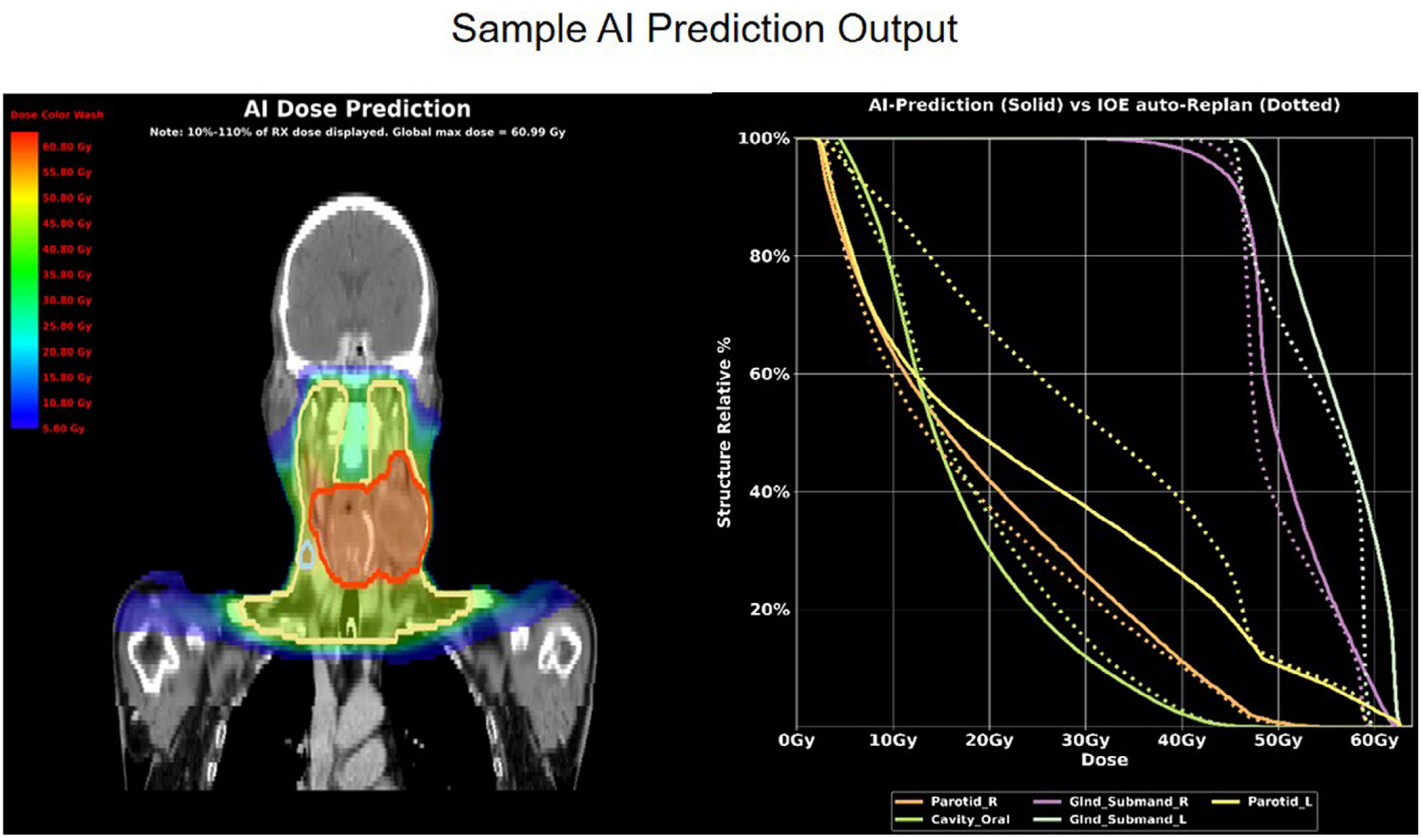
Visual representation of model output. On the left is a coronal view of the AI dose prediction superimposed on a patient image and on the right in a DVH metric comparison for five OAR's.

The focus of this analysis was centered on evaluating 10 organs at risk (OARs) including the PACS, Larynx, mandible, constrictor, ipsilateral parotid, ipsilateral submandibular gland, contralateral parotid, contralateral submandibular gland, oral cavity, and esophagus. For each patient fraction considered in this study, the predicted dose distributions were obtained by running a unique (FT‐PS) model and compared to the original plan dose. An arbitrary dose difference threshold of 1.25 Gy was established for the ten OARs previously discussed, serving as the criterion for initiating a full re‐optimization of the treatment plan using the Ethos system. This threshold was selected as it was considered to represent a sufficiently large dose discrepancy to warrant re‐planning, ensuring that only cases with meaningful potential for improvement in plan quality would undergo the re‐optimization process. Plans where at least one of the OARs showed a mean dose decrease of at least 1.25 Gy were fully re‐optimized by changing the numeric OAR goal in the Ethos planning directive and re‐optimizing the plan within the Ethos system. As mentioned before no new planning goals were added and the priority remained the same as in the original plan. Conversely, plans where none of the ten OARs previously mentioned showed a mean dose decrease of at least 1.25 Gy were excluded from this study. Figure 3, shows a sample output of the dose prediction model. On the right the dose prediction is superimposed on a coronal slice of a patient from this study and on the left there is a DVH metric comparison of five OAR's between the original plan dose distribution and the AI. This data is plotted for visual convenience but the raw dose data coming out of the model was processed using custom Python scripts.

The results of the fully re‐optimized plans were then directly compared to the treated adaptive fraction for comprehensive evaluation. To evaluate the clinical quality of each AI‐guided re‐optimized plan, the plans were exported for a second check using an in‐house clinical calculation system. The analysis focused on plan monitor units (MU) and the relative gamma passing rate to assess plan accuracy and adherence to clinical standards. MU values were used to gauge treatment delivery efficiency and precision, while the relative gamma passing rate provided a quantitative measure of plan conformity with reference dosimetry. Additionally, in a blinded review process, both the original adaptive fraction and the newly optimized plan, guided by dose prediction values, were assessed by a physician for plan preference, ensuring an unbiased evaluation of treatment strategies.

## 3 RESULTS

### 3.1 Dose comparison and OAR sparing

**FIGURE 4:**
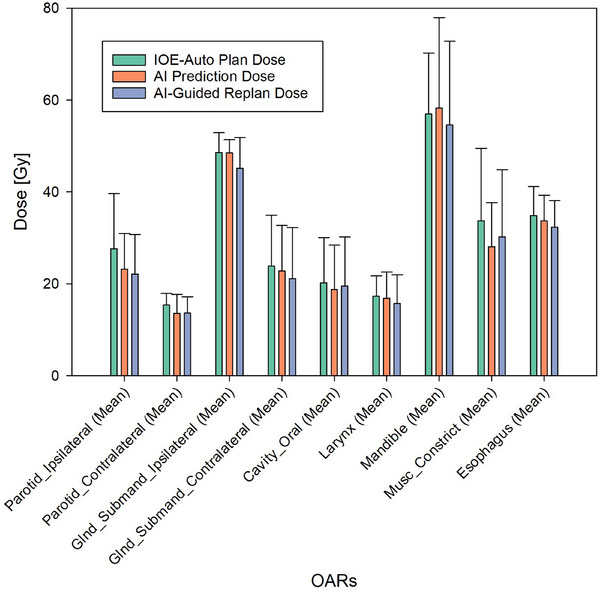
Mean dose for the IOE‐Auto Plan, AI‐Prediction and AI‐guided replan. Nine different OAR's are shown with a color coded legend for plan determination.

Model output from each of the 23 fractions considered in this study were used as a guide for optimization within the Ethos system. For each patient, we compared the AI‐guided re‐optimized plan with the adaptive plan used in the Ethos system, extracting organ‐at‐risk (OAR) doses for our analysis. The AI Prediction represents the initial dose distribution estimated by the model based on the patient's anatomy, used to inform updated planning goals. The AI‐guided replan is the fully re‐optimized plan created within the Ethos system using these updated goals. The IOE‐Auto Plan, by contrast, refers to the standard adaptive plan generated in the Ethos system without incorporating model‐driven updates. Of the 10 organs‐at‐risk (OARs) considered in this study, 9 had at least one case where the dose difference met the threshold criteria of 1.25 Gy, prompting us to adjust the planning goals in the AI‐guided replan. A paired t‐test with a signifance threshold of α = 0.05 of all nine OARs comparing the IOE‐Auto plan and AI Prediction doses resulted in a *p*‐value of 0.0047, indicating a statistically significant difference between these two sets of doses. Similarly, the comparison between the IOE‐Auto plan and AI‐guided Replan doses for all nine OARs showed an even more pronounced difference, with a highly significant *p*‐value of 1.17e‐06. Tables 2 and 3 provide a more in‐depth analysis, including the mean dose difference projected across all fractions of a patient's treatment, the standard deviation, and the associated p‐value. These metrics are presented for comparisons between the IOE‐Auto plan and the AI prediction, as well as between the IOE‐Auto plan and the AI‐guided replan, broken down by organ. Figure 4 shows a series of grouped bar plots meant to visually display the information in Table 2.

**TABLE 2:** Dose Difference projected across all fractions and Standard Deviation results by Organ for IOE‐Auto plan versus AI Prediction and IOE‐Auto plan versus AI Guided replan.

OAR	Mean Dose Difference Gy (IOE‐Auto plan vs. AI Prediction)	Standard Deviation (IOE‐Auto plan vs. AI Prediction)	Mean Dose Difference Gy (IOE‐Auto plan vs. AI Guided replan)	Standard Deviation (IOE‐Auto plan vs. AI Guided replan)
Oral cavity	1.41	1.13	0.63	1.17
Esophagus	1.19	1.7	2.56	1.74
Contralateral submandibular gland	1.05	1.49	2.72	1.95
Ipsilateral submandibular gland	0.1	1.43	3.44	2.37
Larynx	0.44	1.56	1.59	2.01
Mandible	−1.31	6.57	2.35	4.92
Constrictor	5.63	7.94	3.48	4.2
Contralateral parotid	1.82	2.05	1.75	1.88
Ipsilateral parotid	4.41	4.36	5.47	3.46

**TABLE 3:** Total and organ specific p‐values for IOE‐Auto plan versus AI Prediction and IOE‐Auto plan versus AI Guided replan.

OAR	*p*‐value (IOE‐Auto plan vs. AI Prediction)	*p*‐value (IOE‐Auto plan vs. AI Guided replan)	Number of comparisons
Oral cavity	**0.0057**	0.1439	9
Esophagus	0.2536	0.0602	4
Contralateral submandibular gland	0.146	**0.0191**	6
Ipsilateral submandibular gland	0.9372	0.2888	2
Larynx	0.5614	0.1517	5
Mandible	0.7179	0.4092	4
Constrictor	0.0659	**0.0378**	9
Contralateral parotid	0.1745	0.1602	4
Ipsilateral parotid	0.2217	0.1114	3
All OARs	0.0047	1.17e‐06	23

### 3.2 Plan assessment and physician observation study

Table 4 summarizes the plan QA assessment for all fractions in this study, comparing total monitor units (MU) and gamma passing rates between the AI‐guided re‐optimized plans and the original IOE‐Auto plans.^21^ Overall, there were no significant differences in MU values or gamma passing rates between the two plan types. Both MU values and gamma passing rates were consistent across plans, with most gamma passing rates exceeding 98%, indicating strong agreement with reference dosimetry and effective target coverage. These results demonstrate that the AI‐guided plans perform similarly to the IOE‐Auto plans in terms of treatment efficiency and dose accuracy.

**TABLE 4:** Plan QA assessment.

Patient number	Adapted session	AI‐guided IOE plan		IOE‐auto plan	
		Total MU	Gamma pass (%)	Total MU	Gamma pass (%)
Patient 1	18	2373.7	98.58	2213.7	98.77
Patient 1	35	2224.4	98.57	2349.3	97.83
Patient 2	17	2061.7	99.67	2116.2	99.59
Patient 2	34	1999.5	99.69	2112.8	99.54
Patient 3	35	2484.9	97.24	2253.9	97.94
Patient 4	34	2407.8	98.63	2102	99.35
Patient 5	18	1197.5	99.83	1240.8	99.81
Patient 5	35	1320.3	99.9	1190.1	99.9
Patient 6	12	2673.5	99.74	2698.1	99.73
Patient 7	1	2196.6	99.55	2238.3	99.55
Patient 8	28	2687.2	98.66	2734.5	98.48
Patient 8	18	2898.8	98.52	3006.1	97.98
Patient 8	7	2997.6	98.93	2639.8	99.14
Patient 9	20	2582.5	99.59	2707.1	99.34
Patient 9	11	2568.1	99.58	2743.7	99.46
Patient 10	32	2223.4	99.78	2032.4	99.79
Patient 10	23	1982.2	99.81	1985.9	99.82
Patient 11	11	2368.9	99.53	2345.1	99.47
Patient 12	22	2482.5	99.46	2497.9	99.48
Patient 12	11	2568.5	99.46	2650.3	99.34
Patient 13	30	2644.3	99.41	3232.2	98.81
Patient 14	30	2235	99.66	2516.2	99.44
Patient 15	26	3009	98.79	3246.6	98.35

To assess the clinical quality of the AI‐guided re‐optimized plans, a physician reviewed all 23 such plans. For this evaluation, the plans were exported back to the Eclipse treatment planning system and anonymized at the attending physician's request. Each AI‐guided re‐optimized plan underwent meticulous scrutiny to determine its suitability for patient treatment compared to the original plan. Out of the 23 plan comparisons, the physician favored the AI‐guided re‐optimized plans in 19 cases (82.6%), while the original plans were preferred in 4 cases (17.4%). A closer examination of the four cases where the original plans were selected revealed that the lack of significant anatomical changes resulted in very similar initial and re‐optimized plans. These findings highlight the clinical efficacy and quality achieved through AI‐guided re‐optimization.

The blind review process ensured an unbiased evaluation, as the physician was unaware of the plan origins during analysis. The comprehensive assessment considered factors such as target coverage, dose sparing to critical structures, adherence to internal treatment guidelines, and overall plan quality. Specific metrics reviewed included mean and maximum doses to organs‐at‐risk (OARs), dose fall‐off, leakage to surrounding OARs and lower‐dose planning target volumes (PTVs), plan hotspots, and target coverage. The physician's expertise in radiation therapy treatment planning further validated the superiority of the AI‐guided re‐optimized plans.

**FIGURE 5:**
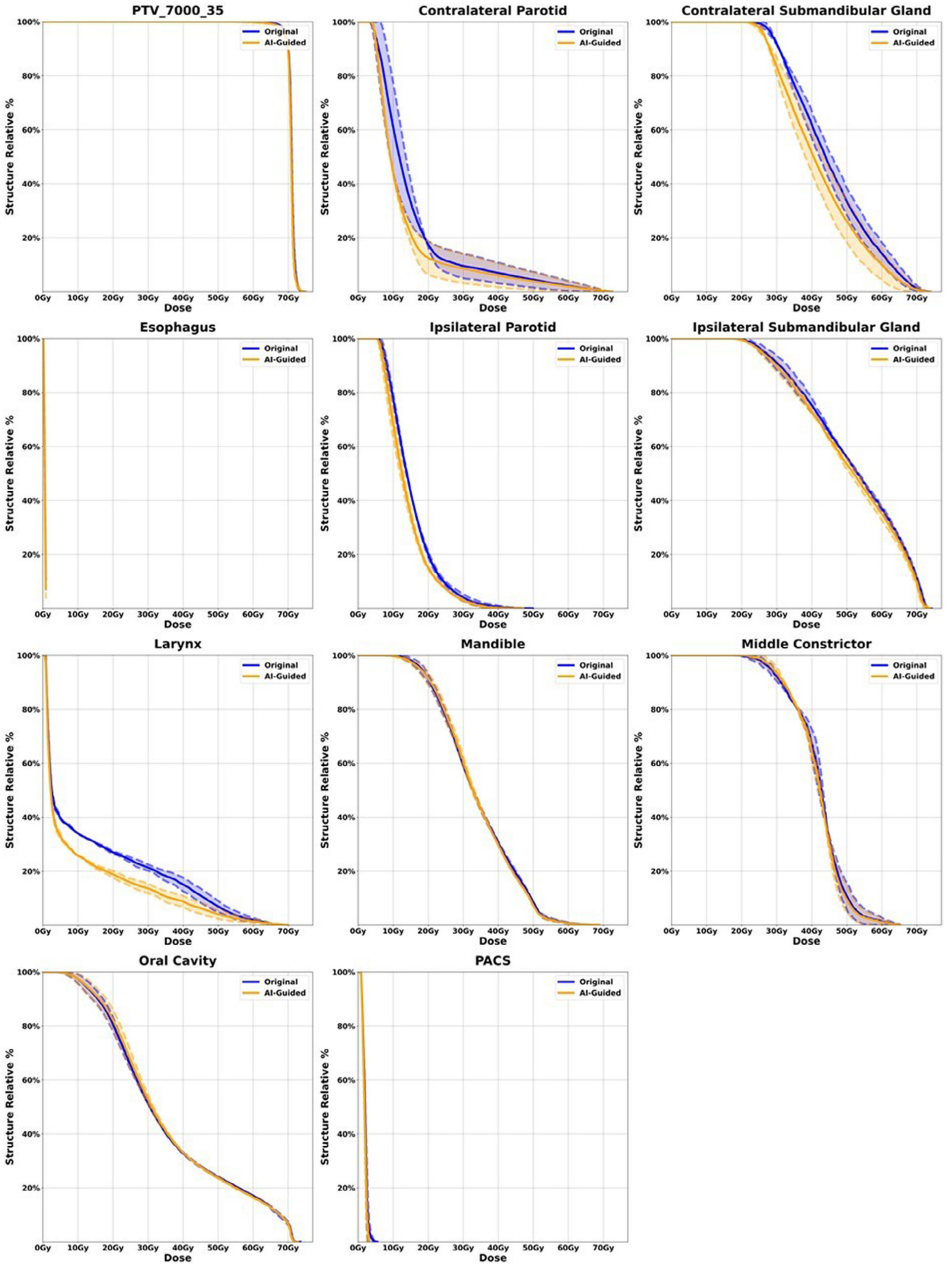
Patient 7 DVH comparison of highest level PTV and high impact OAR's for the original plan versus the AI‐guided re‐plan.

**FIGURE 6:**
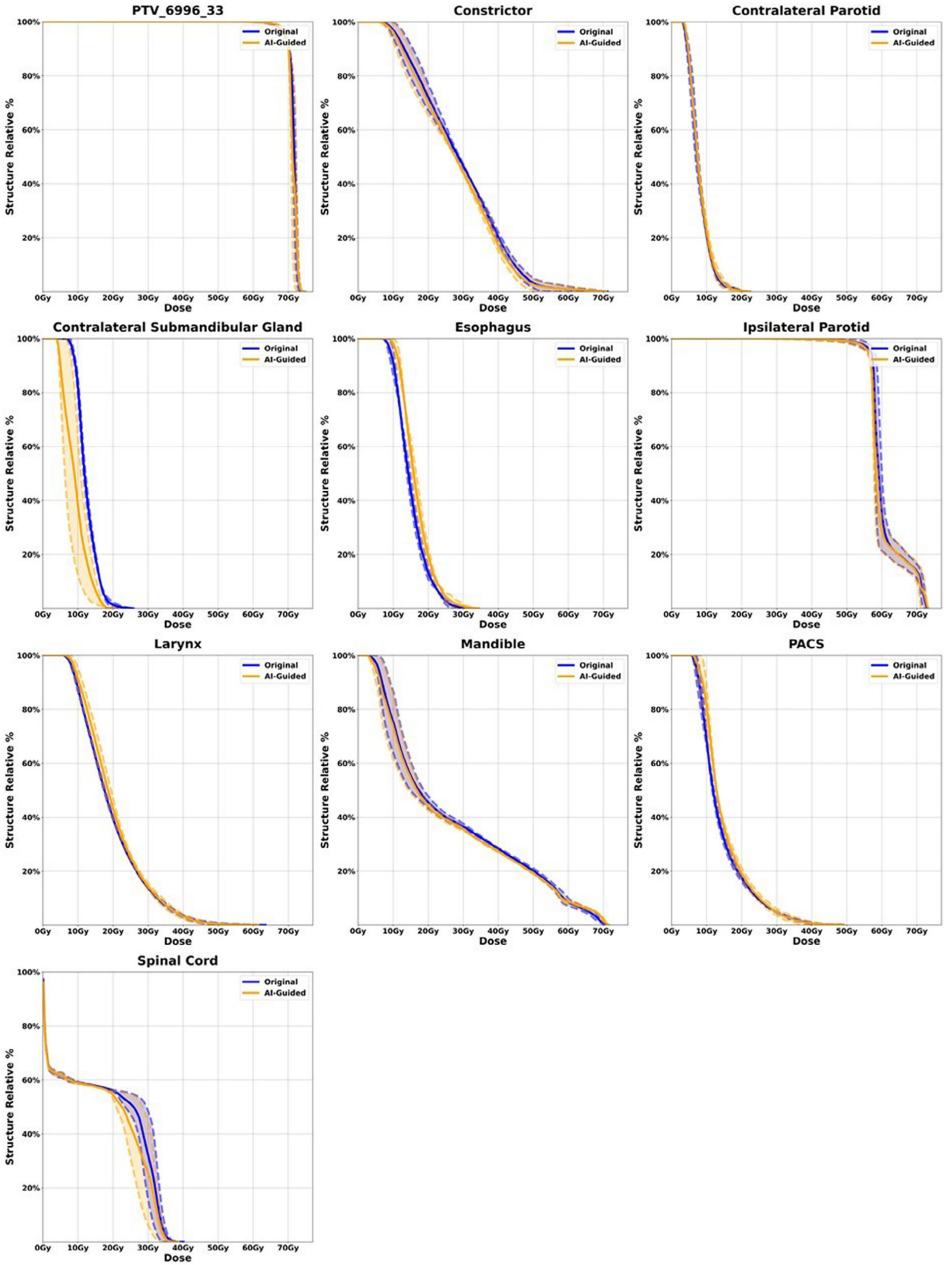
Patient 10 DVH comparison of highest level PTV and high impact OAR's for the original plan versus the AI‐guided re‐plan.

To further illustrate the comparison, patient DVH's for the highest level PTV and a selection of high impact OAR's were plotted for both the original and AI‐guided re‐plan. Patients with more than one fraction were averaged and display a one standard deviation spread. Figures 5 and 6 display the two patients with the most apparent change in patient DVH's. However, plots can be seen for all patients of this study in the supplemental material. Figure 5 demonstrates significant dose savings for the AI‐guided plan compared to the original plan in the contralateral submandibular gland, contralateral parotid gland, and the larynx, with PTV coverage and other OAR sparing remaining virtually unchanged. Similarly, Figure 6 shows dose reductions for the AI‐guided plan in the contralateral submandibular gland and spinal cord, while maintaining comparable coverage for other critical OARs and the PTV.

## 4 DISCUSSION

The findings from this study highlight the potential benefits of incorporating AI‐guided re‐optimization into ART planning. Results show our proposed workflow, although not yet clinically possible on Ethos due to the limitation of the current system, is feasible and could be used during online ART. Additionally, this study highlights the clinical utility of the models and fine‐tuning methodology produced in Maniscaclo et al. While our results only focus on the Ethos system, this method has the potential to be incorporated in any adaptive therapy platform. With the original plan as the baseline for AI model, the prediction can carry over the trade‐off preference of individual patients and dynamically predict the optimal plan quality considering the patient anatomy changes. By doing so, it ensures that each adaptive treatment is tailored to the daily anatomical changes of individual patients.

Adjusting the optimization goal during online adaptive therapy is clinically challenging due to the limited quantitative information to indicate which objective can be further tighten up without significantly trade‐off other plan quality metric. As observed in the dose‐volume histogram (DVH) plots in Figures 5 & 6, it is evident that not all organs at risk (OARs) exhibit significant improvements when the tumor size diminishes. Typically, when tumor shrinkage occurs, it is common to witness notable enhancements in only one or two OARs, rather than all of them. This underscores the importance of implementing an intelligent approach to identify which OARs require goal adjustments and a subsequent push for lower doses, without necessitating a complete re‐plan or redesign of the optimization strategy, which may not always be warranted. This selective approach also enhances our ability to reduce radiation doses to specific OARs without incurring detrimental trade‐offs with others. By focusing our efforts on a few critical OARs and making informed estimates, we can achieve improved treatment outcomes without unnecessarily overhauling the entire optimization process.

**FIGURE 7:**
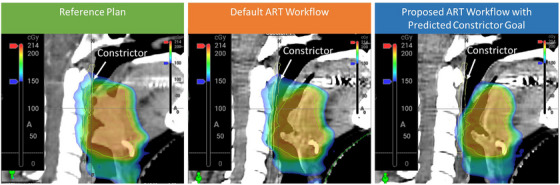
Comparison of treatment adaptation in a prospective clinical case with a prescribed dose of 200 cGy per fraction, contrasting the default workflow, which relies on re‐optimization based on the reference plan's planning goals, with the adapted workflow that employs re‐optimization informed by AI predicted goals.

Figure 7 shows a clinical example of a prospective case adapted based on the proposed workflow. The physician observed the tumor shrunk away from the constrictor and requested online adaptive treatment. The first attempt of adaptation was based of the default workflow, optimizing with the reference planning goal. Given the clinical system does not allow real‐time adjustment of goal, we performed the adaptive dose prediction based on the contour of the first attempt and updated the constrictor goal to a lower number while other metric were kept the same in the Ethos TPS for the next ART. The second attempt was made the next day based on the proposed workflow. It is demonstrated the updated goal can be reasonably achieved and exhibited in higher plan quality.

The statistical analysis across the nine OAR's directly adjusted during retrospective re‐optimization show significant differences in dose values. Both the comparisons between the IOE‐Auto plan and AI Prediction doses, as well as between the IOE‐Auto plan and AI‐guided replan doses, resulted in *p*‐values below the threshold of α = 0.05 for statistical significance. Table 3 further analyzes these results by individual organ. From these results only a select few organs including oral cavity, contralateral submandibular gland and the constrictor show *p*‐value significance. This is almost certainly due to the limited number of comparisons when we analyze our results on an organ‐by‐organ basis. This is an unfortunate consequence of the limited selection of patients available for this study and is recognized as a limitation of this work. It is the intention of this group to conduct follow‐up studies with larger and more diverse selection of patients and cancer sites.

Referencing Figure 4 for the parotid glands, submandibular glands, oral cavity and larynx, and esophagus both the average AI prediction dose and average AI‐guided re‐plan dose consistently show lower values than the average IOE‐ Auto plan dose, indicating successful dose reduction achieved through AI‐guided re‐optimization. In addition, the average AI‐guided re‐plan dose is the same or lower than the average AI prediction dose. This indicates the ability of the TPS optimizer to effectively provide the dose sparing for these OARs evident in the AI prediction dose. An interesting trend is observed with the mandible and muscle constrictor. For the mandible, the average AI prediction dose is slightly higher than the original plan dose, but the average AI‐guided re‐plan dose shows a reduction. This indicates that the optimizer pushed harder to spare this structure than what was asked for in the planning directive. For the muscle constrictor we see the exact opposite, the average AI‐ prediction dose is slightly lower than the average AI‐guided re‐plan dose. This indicates that the optimizer didn't push the structure hard enough to achieve the values we asked for in the planning directive. However, the values are still lower than the average IOE‐ Auto plan dose. In summary, the data show two main things: consistent dose reduction achieved by AI‐guided re‐optimization in most OARs featured in this study, and the consistent ability of the Ethos system to achieve the AI prediction dose values put into the planning directive during re‐optimization. This highlights the potential of AI‐guided re‐optimization in improving plan quality and sparing critical structures in ART planning. Table 4 also emphasizes that using AI to guide re‐optimization does not result in signifcant changes to plan MU or QA results.

Table 2 explicitly quantifies the average total dose savings seen by the AI‐ guided re‐plan versus the IOE‐Auto plan. Dose savings vary from 0.63 Gy for the Oral cavity to 5.47 Gy for the ipsilateral parotid gland. Considering these dose reductions, this methodology has great potential to minimize acute and long‐term side effects, improve treatment tolerability, and enhance patient quality of life during and after radiation therapy. The significant dose savings achieved in the parotid glands are particularly noteworthy as they are highly susceptible to radiation‐induced damage, leading to xerostomia and related complications. Furthermore, the dose savings observed in the submandibular glands, oral cavity, larynx, mandible, and constrictor muscles, and esophagus have important clinical implications. Preservation of these structures is crucial for maintaining normal swallowing function, speech quality, and jaw mobility. Minimizing radiation‐induced damage to the larynx and esophagus is particularly beneficial for patients undergoing HN radiation therapy, as it can reduce the risk of dysphagia and improve swallowing function. Additionally, sparing the mandible and constrictor muscles can help prevent radiation‐induced bone damage and reduce difficulties in mastication and speech.

The results of physician guided analysis for all 23 fractions show that 19 out of 23 plans chosen over the original adapted session plans. This preference demonstrates not only the clinical acceptability of the AI dose prediction but the enhancement of the overall plan quality. This is a very important result as this clearly demonstrates the potential impact of AI‐guided re‐optimization in ART planning. While the results for all patients are provided in the supplementary material, we have chosen to display patients 7 and 10 in Figures 5 and 6, respectively, because these cases clearly showcase the dose savings achieved by the AI‐guided plans in terms of DVH. Looking at Figure 5 we can see significant dose savings for the AI‐guided plan compared to the original plan for the contralateral submandibular gland, control parotid gland and the larynx. PTV coverage and other OAR sparing is virtually the same. Figure 6 shows dose savings from the AI‐guided plan compared to the original plan for the contralateral submandibular gland and spinal cord while providing very similar coverage for other high impact OAR's as well as PTV coverage.

In the context of this work, it is vital to emphasize the broader implications and potential applications of the finely tuned patient‐specific (FT‐PS) deep‐learning dose prediction model introduced in this study. This tool can serve as a valuable decision support tool for adaptive therapy not only within the context of Ethos but also for other treatment planning systems like Eclipse, which utilize similar optimization algorithms. The model can also be transfer learned to various adaptive therapy systems such as MR‐guided adaptive therapy. One significant advantage of this FT‐PS model is its adaptability to different clinical settings, including both ART and regular linear accelerators. Institutions can define their own adaptation thresholds considering available resources, patient‐specific factors, and clinical goals. This flexibility empowers healthcare providers to make informed decisions about when to adapt treatment plans based on their specific capabilities and priorities. Moreover, it's worth noting that the study successfully demonstrated the feasibility of meeting the predicted adaptation needs. This finding provides strong support for the practicality and reliability of implementing this AI‐guided approach in clinical practice.

While the results are encouraging, the study's focus on head and neck cancer patients limits the broader applicability of the findings. Expanding this methodology to other treatment sites is critical to fully evaluate its potential impact. Future research should prioritize testing and refining this approach across diverse cancer types to ensure its generalizability and relevance to a wider range of clinical scenarios. In order to expand to other treatment sites, additional models will need to be made and clinically evaluated prior to use. In addition, different treatment facilities should be explored to validate this approach as well. The inclusion of a larger and more diverse patient cohort, including different tumor sites and treatment techniques, would help validate the effectiveness of AI‐guided re‐optimization across a broader range of cases and facilities. Additionally, the integration of AI‐guided re‐optimization into clinical workflows requires careful consideration, particularly when dealing with closed systems. Accessing and effectively incorporating AI‐driven processes into these proprietary environments can pose certain difficulties. Collaboration and communication between radiation oncologists, medical physicists, and AI experts are crucial for ensuring optimal treatment planning outcomes without significantly increasing the time the patient is on the table.

## 5 CONCLUSIONS

This study underscores the significant potential of AI‐guided re‐optimization in enhancing adaptive radiotherapy (ART) planning. By demonstrating dose reductions in critical structures and optimizing the balance between target coverage and organ‐at‐risk sparing, AI algorithms can improve treatment outcomes and personalize cancer care. Our results confirm the feasibility of integrating AI‐guided re‐optimization into online ART workflows, even though current limitations prevent its clinical implementation on the Ethos system. Importantly, the methodology developed by Maniscalco et al. proves clinically useful, and while our focus was on the Ethos system, this approach could be applied to any adaptive therapy platform. However, it is crucial to acknowledge that further research is needed to establish the generalizability, long‐term benefits, and cost‐effectiveness of AI‐guided re‐optimization. Larger‐scale studies encompassing diverse patient cohorts and long‐term follow‐up are necessary to validate the effectiveness and robustness of this approach across different clinical scenarios. Additionally, studies evaluating the resource utilization and practicality of implementing AI‐guided re‐optimization are essential for its integration into routine clinical practice.

## AUTHOR CONTRIBUTIONS

Mu‐Han Lin, Dan Nguyen and Austen Maniscalco designed this study. Sean Domal performed the data collection and analysis, figure generation and drafted the first iteration of the manuscript. Mu‐Han Lin provided clinical supervision and input to this project. All authors revised and approved the final manuscript.

## CONFLICT OF INTEREST STATEMENT

The authors declare no conflicts of interest.

## Data Availability

The data that support the findings of this study are available from the corresponding author upon reasonable request.
